# Trained Innate Immunity by Repeated Low-Dose Lipopolysaccharide Injections Displays Long-Term Neuroprotective Effects

**DOI:** 10.1155/2020/8191079

**Published:** 2020-10-01

**Authors:** Xiao-yan Zhou, Rong Gao, Jian Hu, Da-peng Gao, Yan-ling Liao, Jian-jun Yang

**Affiliations:** ^1^Department of Anesthesiology, Jinling Hospital, Jinling Clinical Medical College of Nanjing Medical University, Nanjing, Jiangsu, China; ^2^Department of Emergency and Intensive Care Medicine, Nanjing Integrated Traditional Chinese and Western Medicine Hospital, Affiliated with Nanjing University of Chinese Medicine, Nanjing, Jiangsu, China; ^3^Department of Anesthesiology, Nanjing Lishui People's Hospital, Nanjing, Jiangsu, China; ^4^Department of Anesthesiology, Pain and Perioperative Medicine, The First Affiliated Hospital of Zhengzhou University, Zhengzhou, Henan, China

## Abstract

Disrupted immune response is an important feature of many neurodegenerative conditions, including sepsis-associated cognitive impairment. Accumulating evidence has demonstrated that immune memory occurs in microglia, which has a significant impact on pathological hallmarks of neurological diseases. However, it remains unclear whether immune memory can cause subsequent alterations in the brain immune response and affect neurobehavioral outcomes in sepsis survivors. In the present study, mice received daily intraperitoneal injection of low-dose lipopolysaccharide (LPS, 0.1 mg/kg) for three consecutive days to induce immune memory (immune tolerance) and then were subjected to sham operation or cecal ligation and puncture (CLP) 9 months later, followed by a battery of neurobehavioral and biochemical studies. Here, we showed that repeated low-dose LPS injection-induced immune memory protected mice from sepsis-induced cognitive and affective impairments, which were accompanied by significantly decreased brain proinflammatory cytokines and immune response. In conclusion, our study suggests that modulation of brain immune responses by repeated LPS injections confers neuroprotective effects by preventing overactivated immune response in response to subsequent septic insult.

## 1. Introduction

Accumulating evidence has demonstrated that sepsis is a pivotal component in mediating cellular senescence, changes in neuronal plasticity, neuronal loss, and consequent cognitive impairments [1–3]. Animal studies have also shown depressive-like behavior and learning and memory impairments after sepsis by lipopolysaccharide (LPS) challenge or cecal ligation and puncture (CLP) [4, 5]. However, there is currently no effective preventive treatment for sepsis-associated cognitive impairments.

Immune memory is a well-known phenomenon in the adaptive immune system, which provides long-lasting protection against reinfection in case of similar pathogen [6]. Intriguingly, immune memory also occurs in microglia and significantly affects pathological hallmarks of neurological disease in mouse models [7]. Dysregulated activation of trained immunity can lead to either hyperinflammation or immunodepression, indicating immune memory in microglia is highly plastic [8]. In one previous study, it has been shown that a single LPS injection induced immune training 1 day later, whereas repeated LPS injections for 4 consecutive days caused immune tolerance in the brain, as reflected by increased and decreased brain cytokine levels, respectively [8]. One recent study has also shown that restoring the delicate balance of immune homeostasis confers substantial protection against stress-induced anxiety- and depression-like behaviors [9]. In view of the detrimental role of sepsis and the absence of effective therapies, studies based on immune memory modulation are urgently needed.

In the brain, immune memory has been studied on relatively short stage in most disease disorders [9]; it is necessary to elucidate whether immune memory confers long-term protection against behavioral deficits. Therefore, we tested the hypothesis that repeated LPS injections can induce long-term immune memory and displays neuroprotective effects in response to subsequent septic insult.

## 2. Materials and Methods

### 2.1. Animals

**Figure 1:**
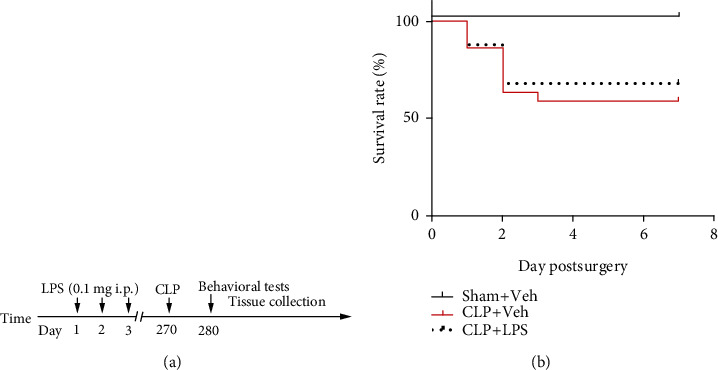
Timeline of the experimental procedure in mice (a). Effects of CLP and repeated LPS injections on survival rate (b). LPS: lipopolysaccharide; CLP: cecal ligation and puncture; Veh: vehicle.

All animal use procedures were approved by the Committees at Jinling Clinical Medical College of Nanjing Medical University, Nanjing, China, and were in accordance with the Care and Use of Laboratory Animals approved by the National Institutes of Health of the United States. Eighty male C57BL/6 mice (3-4 months) were purchased from the Animal Center of Jinling Hospital, Nanjing, China. To exclude the influence of estrogen cycle on immune response, only male mice were used in the present study. The mice were housed 4-5 per cage on a 12 h light–dark cycle in a room of 24 ± 1°C with food and water available *ad libitum*. Before the experimental study, animals were allowed to acclimatize for at least one week. The experimental protocol in the present study is shown in Figure 1(a).

### 2.2. Immunity Memory Induced by Repeated Low-Dose LPS Injections

LPS (from *Escherichia coli* 0111:B4, Sigma, St. Louis, MO, Shanghai, China) was dissolved in 0.9% NaCl. All injections were prepared fresh and given intraperitoneally (*i.p.*) for three consecutive days in a final injection volume of 10 ml/kg body weight between 10 a.m. and 12 a.m. The LPS dosage of 0.1 mg/kg and duration of treatment were selected because this protocol was able to induce microglia memory with minimal effect on locomotor activity [8]. To minimize the animals used, mice in the sham + vehicle group was served as the control group.

### 2.3. Animal Model of Sepsis

Sepsis was induced by CLP with minor modifications as we previously described [5]. This model allows the release of fecal material into the peritoneal cavity to generate an exacerbated immune response, which clinically mimics human sepsis. Under a sterile condition, about 1 cm abdominal midline incision was performed to expose the cecum under aseptic conditions. The cecum was ligated with a 3.0 silk suture 0.6 cm from the distal end and then perforated twice with a 22-gauge needle until a small amount of feces squeezed through the puncture site. The intestinal canal was then situated back in the abdomen, and the incision was sutured with a sterile 3.0 silk. Mice were injected with 0.9% saline solution (subcutaneously, 20 ml/kg of body weight) immediately after the CLP and then returned to their cages. In the sham group, animals underwent similar surgical procedures, but the cecum was neither ligated nor perforated. To keep the model stable, all the procedures were performed by one investigator who was blind to the animal grouping.

### 2.4. Behavioral Experiments

A battery of well-established behavioral tests was used to assess behavioral alterations as previously described [10]. All behavioral studies were performed between 11:00 a.m. and 17:00 p.m. under dim lighting conditions. All behavioral experiments were recorded by a video camera (XR-XZ301, Shanghai Softmaze Information Technology Co. Ltd., Shanghai, China). The chamber was cleaned with 75% ethanol after each test.

### 2.5. Open Field Test

To address the possible effects of repeated LPS injection and CLP on locomotor activity, mice were evaluated in the open field test. The apparatus consists of a white polyester resin chamber (40 cm × 60 cm × 50 cm). The mice were placed in the center of the arena and allowed to explore for 5 min, and the total distance moved and the time spent in the center were recorded.

### 2.6. Elevated Plus Maze

The anxiolytic-like behavior of mice was evaluated by the elevated plus maze test. The elevated plus maze consists of a central area of diameter 10 cm, from which four arms extended of length 45 cm and width 5 cm. Two arms were open without walls, while the other two were enclosed by high walls. For this test, the mouse was placed in the center of the apparatus, and the number of entries and time spent in the open arms was scored in a 5 min session.

### 2.7. Sucrose Preference Test

Mice were placed individually with a two bottles, one with 1% sucrose solution and the other containing tap water. Mice were then given a free choice between either tap water or 1% sucrose in tap water solution. Twenty-four hours later, the bottles were then weighed to measure how much liquid was consumed. The sucrose preference (%) = sucrose consumption/(sucrose consumption + water consumption) × 100.

### 2.8. Fear Conditioning Test

The mouse was placed in the conditioning chamber for 3 min as an accommodation period, and then, one tone-foot-shock pairing (tone, 30 s, 65 dB, 1 kHz; foot-shock, 2 s, 0.75 mA) was delivered. The mouse was allowed to explore the chamber for another 30 s after the shock. After 24 h, the contextual fear conditioning test was assessed to evaluate that hippocampus-dependent memory was performed by placing the mice in the same chamber again without any stimulation. The tone fear conditioning test was assessed 2 h after the contextual fear conditioning test in a novel chamber changed in shape, color, and smell, and the training tone was delivered for 3 min.

### 2.9. Forced Swim Test

In the forced swim test, mice were forced to swim individually in an acrylic cylinder (height: 30 cm; diameter: 15 cm; depth: 15 cm) filled with water (22–24°C) for 6 min. The immobility was scored in the final 4 min only. Time spent immobile (absence of movement except leg kicks to stay afloat) is then used as a measure of behavioral despair and helplessness, a rodent analogue of depressive-like behavior.

### 2.10. Inflammatory Cytokine Measurements

Mice were killed by an injection of 2% sodium pentobarbitone (50 mg/kg, *i.p.*), and the prefrontal cortex (PFC) and hippocampus were collected, then separated, and placed in a homogenizer. Homogenates were centrifuged at 10,000 g for 10 min at 4°C. Cytokines including TNF-*α*, IL-1*β*, IL-2, IL-4, IL-5, IL-6, IL-10, IL-12p70, IFN-*γ*, and KC/GRO were measured by the V-Plex Proinflammatory Cytokine ELISA kit (Meso Scale Discovery, QuickPlex SQ120) according to the manufacturer's instructions.

At the indicated time-points, mice were deeply anaesthetized by 2% sodium pentobarbitone (50 mg/kg, *i.p.*); blood was collected from the right ventricle of the heart which was centrifuged at 10,000 g for 10 min at 4°C. For serum cytokine measurement, we performed serum levels of inflammatory cytokines by using the ELISA kits for rats according to the manufacturers' instructions (TNF-*α* (Abcam, ab212073), IL-1*β* (Abcam, ab197742), IL-6 (Abcam, ab213462)). Values were expressed as pg/ml.

### 2.11. Western Blotting

The hippocampal samples were lysed, and protein concentrations were determined as we described previously [1]. Equivalent amounts of proteins (10 *μ*g, 1 *μ*g/*μ*l) per lane were separated on SDS-PAGE gels and then transferred to polyvinylidinene fluoride membranes. Members were blocked with 5% skimmed milk in Tris Buffered Saline with Tween (TBST) for 1 h at room temperature. And then, the members were incubated at 4°C overnight with primary antibodies including mouse anti-IL-1 beta (Abcam, Cambridge, UK, 1 : 1000), mouse NF-*κ*B p65 (phospho S536, Abcam, 1 : 1000), rabbit anti-IFN-*γ* (APExBIO, Houston, USA, 1 : 1000), mouse anti-IL-6 (APExBIO, 1 : 1000), and anti–GAPDH (Proteintech, 1 : 10000). After washing in TBST for three times, the members were incubated 1 h at room tempreture with IgG-horseradish peroxidase-conjugated secondary antibody (Servicebio, 1 : 3000). The protein bands were detected by enhanced chemiluminescence, exposed onto X-ray film, and quantitated with the Image J software (National Institutes of Health, Bethesda, MD, USA).

### 2.12. Immunofluorescence

Under deep anesthesia, animals were perfused transcardially with normal saline, followed by 4% paraformaldehyde in phosphate buffered saline. The brains were harvested and postfixed in 4% PFA overnight and were gradient eluted using 10%, 20%, and 30% sucrose and then imbedded with OCT and stored at -80°C. The brains were freeze-mounted in optimal cutting temperature embedding medium, cut into 25 *μ*m thick sections using a cryostat, and mounted on slides. Slices were blocked with 3% bovine serum albumin for 1 hour at room temperature. The sections were then incubated with a rat anti-ionized calcium-binding adaptor molecule-1 (IBA1) (Abcam, Cambridge, UK, 1 : 200) antibody overnight at 4°C, followed by 1 hour incubation with the secondary antibodies Cy3-conjugated donkey anti-rat IgG (1 : 300; Santa Cruz Biotechnology, Dallas, TX) at room temperature. After washing in PBS, sections were counterstained with 4′, 6-diamidino-2-pheny-lindole and mounted on glass slides and coverslipped with fluorescence mounting medium. The intensity of IBA1 and GFAP positive cells was obtained using fluorescence microscopy (Leica Microsystems, Wetzlar, Germany).

### 2.13. Statistical Analysis

Statistical analysis was performed by GraphPad Prism 7.0 (GraphPad Software, La Jolla, CA, USA). Data are presented as mean ± SEM. All presented data were assessed for normal distribution by the Kolmogorov–Smirnov test. Differences among multiple groups were tested using a one-way analysis of variance test for normal distribution data or a nonparametric Kruskal–Wallis test for a nonnormal distribution where appropriate. The survival rate was estimated by the Kaplan–Meier method and compared by the log–rank test. A *P* < 0.05 was considered statistically significant.

## 3. Results

### 3.1. Survival Rate following CLP

As shown in Figure 1(b), no animal died in the sham + vehicle group. The survival rate in the CLP + vehicle and CLP+ LPS group was 59% and 68%, respectively.

### 3.2. Behavioral Changes following CLP

**Figure 2:**
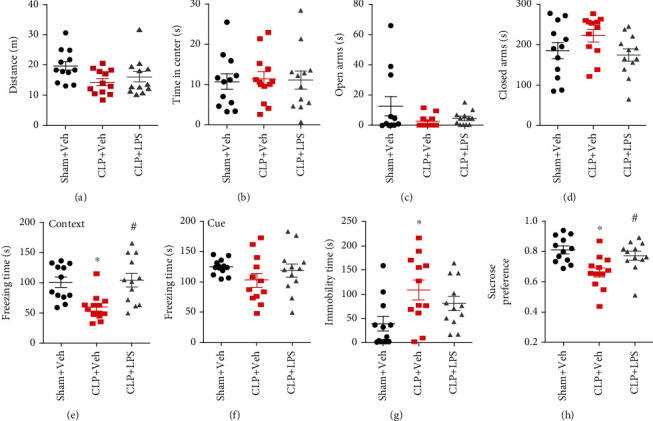
Effects of repeated LPS injections and CLP on neurobehavioral outcomes. CLP induced a significantly decreased freezing time to context, immobility time, and sucrose preference which were observed in the CLP group, whereas repeated LPS injections prevented the decreased freezing time to context and sucrose preference. Data represent mean ± SEM; *n* = 12/group. ^∗^*P* < 0.05 vs. sham + Veh group; #*P* < 0.05 vs. CLP + Veh group. LPS: lipopolysaccharide; CLP: cecal ligation and puncture; Veh: vehicle.

The open field test was performed to investigate whether sepsis influences locomotor activity and anxiety-like behavior. As shown in Figures 2(a) and 2(b), there was no difference in total distance traveled and time spent in the center of the open field arena among the groups (all *P* > 0.05), which suggested that animals recovered from sepsis development. In the elevated plus maze test, no difference in the time spent in the open and closed arms was observed among the groups (Figures 2(c) and 2(d), all *P* > 0.05).

We then performed the fear conditioning tests to evaluate whether sepsis affected contextual fear memory. As shown in Figures 2(e) and 2(f), CLP significantly decreased freezing time to context relative to that of sham + vehicle mice, which was prevented by repeated LPS injections (*F*_2,33_ = 7.922, *P* = 0.0015). However, CLP did not affect freezing time in the auditory-cued fear test when compared with the sham + vehicle group (*F*_2,33_ = 1.424, *P* = 0.2551).

In the forced swim test, mice in the CLP + vehicle group showed an evident increase in immobility time as compared with the sham + vehicle mice, which was not reversed by repeated LPS injections (*F*_2,33_ = 4.548, *P* = 0.018, Figure 2(g)). However, sepsis mice displayed significantly less preference for sucrose relative to that of sham + vehicle mice in the sucrose preference test, which was prevented by repeated LPS injections (*F*_2,33_ = 7.166, *P* = 0.0026, Figure 2(h)).

### 3.3. Serum Cytokine Changes following LPS Injection

**Figure 3:**
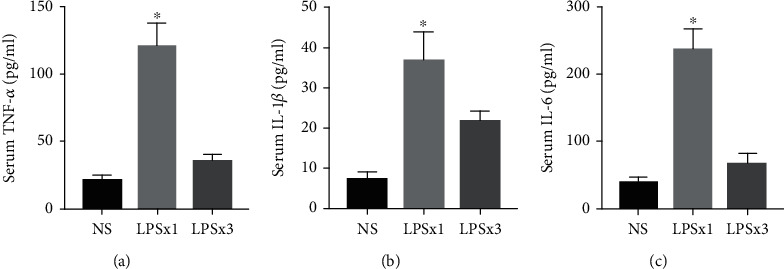
Serum cytokine changes following LPS injection. ELISA showed increased TNF-*α*, IL-1*β*, and IL-6 levels following one dose of LPS injection. However, repeated LPS injections abolished TNF-*α*, IL-1*β*, and IL-6 production. Data represent mean ± SEM; *n* = 6/group. ^∗^*P* < 0.05 vs. NS group. LPS: lipopolysaccharide; NS: normal saline.

To determine the effects of LPS injections on serum cytokine changes, we gave animals daily of LPS (*i.p.*) on three consecutive days. As shown in Figure 3, one dose of LPS induced significantly increased serum TNF-*α* (Kruskal–Wallis statistic = 13.05, *P* < 0.0001), IL-1*β* (Kruskal–Wallis statistic = 12.04, *P* = 0.0002), and IL-6 (Kruskal–Wallis statistic = 12.8, *P* < 0.0001) levels 6 h after injection. However, repeated LPS injections abolished TNF-*α*, IL-1*β*, and IL-6 production. These results suggested that repeated LPS injections induced immune tolerance.

### 3.4. Brain Cytokine Changes following CLP

**Figure 4:**
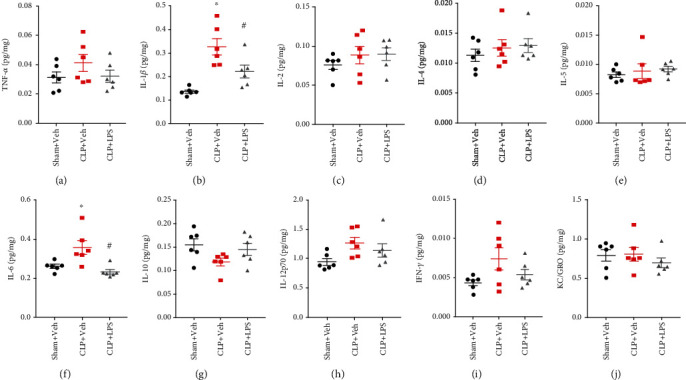
Effects of repeated LPS injections and CLP on hippocampal cytokines. Tissue ELISA showed increased IL-1*β* and IL-6 levels in the hippocampus following CLP, which were prevented by repeated LPS injections. Data represent mean ± SEM; *n* = 6/group. ^∗^*P* < 0.05 vs. sham + Veh group; #*P* < 0.05 vs. CLP + Veh group. LPS: lipopolysaccharide; CLP: cecal ligation and puncture; Veh: vehicle.

**Figure 5:**
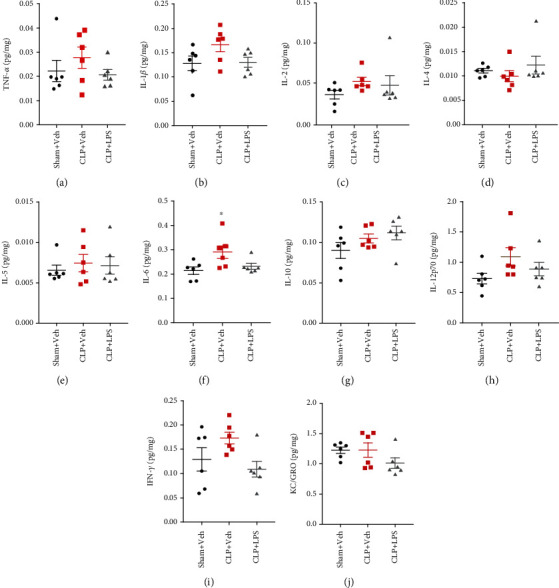
Effects of repeated LPS injections and CLP on prefrontal cortex cytokines. Tissue ELISA showed the increased IL-6 expression in the prefrontal cortex following CLP, which was not prevented by repeated LPS injections. Data represent mean ± SEM; *n* = 6/group. ^∗^*P* < 0.05 vs. sham + Veh group; #*P* < 0.05 vs. CLP + Veh group. LPS: lipopolysaccharide; CLP: cecal ligation and puncture; Veh: vehicle.

Figures 4 and 5 indicate changes in hippocampal and PFC inflammatory mediator expressions following CLP, respectively. In the hippocampus, increased IL-1*β* and IL-6 levels were observed following CLP. However, repeated LPS injections were able to decrease IL-1*β* (*F*_2,15_ = 10.23, *P* = 0.0006) and IL-6 (*F*_2,15_ = 8.682, *P* = 0.0034) levels as compared with the CLP + vehicle group. There was no difference in TNF-*α*, IL-2, IL-4, IL-5, IL-10, IL-12p70, IFN-*γ*, and KC/GRO levels among these groups (all *P* > 0.05). There were significantly increased IL-6 (*F*_2,15_ = 4.472, *P* = 0.03) level in the PFC following CLP, which was not prevented by repeated LPS injections. There was no difference in TNF-*α*, IL-1*β*, IL-2, IL-4, IL-5, IL-10, IL-12p70, IFN-*γ*, and KC/GRO levels among these groups (all *P* > 0.05).

**Figure 6:**
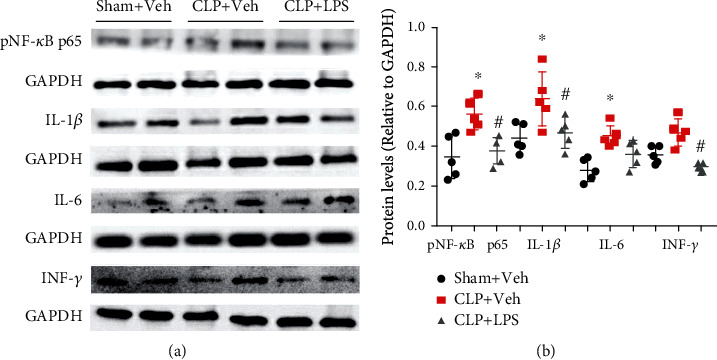
Effects of repeated LPS injections and CLP on hippocampal inflammatory mediators. There were significantly increased hippocampal pNF-*κ*B p65, IL-1*β*, and IL-6 levels following CLP. However, repeated LPS injections were able to prevent the increased pNF-*κ*B p65 and IL-1*β* levels. Data represent mean ± SEM; *n* = 5/group. ^∗^*P* < 0.05 vs. sham + Veh group; #*P* < 0.05 vs. CLP + Veh group. LPS: lipopolysaccharide; CLP: cecal ligation and puncture; Veh: vehicle.

We further used western blotting to confirm the above results. As shown in Figure 6, there were significantly increased hippocampal IL-1*β* levels (*F*_2,12_ = 11.66, *P* = 0.0015) and IL-6 (*F*_2,12_ = 15.41, *P* = 0.0005) following CLP, while repeated LPS injections can prevent increased IL-1*β* but not IL-6. In addition, we showed that pNF-*κ*B p65 was significantly increased after CLP, which was also prevented by repeated LPS injections (*F*_2,12_ = 8.86, *P* = 0.0043). Overall, these results were consistent with most of the ELISA results.

### 3.5. Brain Microglia Changes following CLP

**Figure 7:**
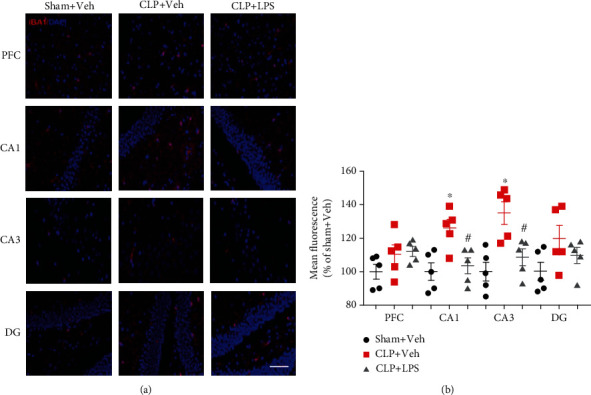
Effects of repeated LPS injections and CLP on IBA1-positive cells. Immunohistochemistry showed increased intensity of IBA1-positive in the CA1 and CA3 following CLP, which were partially prevented by repeated LPS injections. Data represent mean ± SEM; *n* = 5/group. ^∗^*P* < 0.05 vs. sham + Veh group; #*P* < 0.05 vs. CLP + Veh group. LPS: lipopolysaccharide; CLP: cecal ligation and puncture; Veh: vehicle; PFC: prefrontal cortex; CA1: cornu ammonis 1; CA3: cornu ammonis 3; DG: dentate gyrus. Red indicates IBA1; blue indicates DAPI; scale bar = 100 *μ*m.

To assess the status of microglia following CLP, we measured ionized calcium binding adapter molecule (IBA1) for microglia in the hippocampus or PFC after neurobehavioral tests. As shown in Figure 7, there was no increased intensity of IBA1 cells in the PFC following CLP (*F*_2,12_ = 2.158, *P* = 0.1583). In the hippocampus, CLP significantly upregulated the intensity of IBA1-positive cells in the CA1 and CA3 compared with the sham + vehicle group. Repeated LPS injections significantly decreased the intensity of IBA1 cells in the CA1 (*F*_2,12_ = 7.828, *P* = 0.0067) and CA3 (*F*_2,12_ = 10.24, *P* = 0.0025) as compared with the CLP + vehicle group.

## 4. Discussion

In the present study, we showed that repeated low-dose LPS stimuli-induced immune memory has a long-lasting impact on microglia memory, leading to a decreased response to a subsequent sepsis insult and consequent cognitive improvement.

Disrupted immune response is an important feature of many neurodegenerative conditions, including sepsis-associated cognitive impairment. Thus, immunotherapy becomes one of the most exciting therapeutic strategies and receives increasing attention [7]. Indeed, it has been demonstrated that neutralization of proinflammatory mediators such as TNF-*α* and IFN-*β* during the hyperinflammation phase of sepsis and boost suppressed immunity with IFN-*β* during the immunosuppression phase both showed therapeutic effects [11]. Trained immunity may exert therapeutic benefits for many conditions, including cancer and sepsis, whereas actively suppressing trained immunity may also be helpful for autoimmune disorders and cardiovascular diseases [12–15]. These results suggested that understanding of the individual immune state will help with the effort to develop more effective approaches for treating dysregulated inflammatory disorders while taking into account the positive aspects of this phenomenon.

Recent studies have shown that immune memory occurs in microglia, which has a significant impact on pathological hallmarks of neurological diseases [8]. Immune memory has two forms: immune training and immune tolerance, which refers to enhanced or suppressed immune responses upon secondary stimulation, respectively. Indeed, one recent study investigating the long-term effects of single and repeated LPS treatments suggests that immune training- and tolerance-inducing LPS stimuli were sufficient to modulate brain pathology in mouse models of stroke or Alzheimer's disease pathology when applied weeks to months before pathology onset [8]. These findings indicate that microglia may have either exaggerated or decreased responsiveness to subsequent inflammatory stimuli, leading to either beneficial or harmful outcomes. Thus, restoring the delicate balance of immune homeostasis is critical for the treatment of sepsis-associated cognitive impairments. In view of the persistently high mortality associated with sepsis and the absence of effective therapies, advances based on modulating the immune response are urgently needed. However, the effects of trained immune memory long before severe sepsis have not been explored.

Increasing evidence indicates that endotoxin tolerance is an essential immune-homeostatic response to repeated exposures to LPS that induces a state of altered responsiveness in macrophage, resulting in repression of the proinflammatory gene expression and increased expression of factors that mediate the resolution of inflammation [16–18]. In the present study, we showed that immune memory can last for up to 9 months and exert beneficial effects against subsequent sepsis insult, as reflected by decreased cytokine expressions. Consistently, there are many reports regarding the protective effect of LPS preconditioning on the destructive stimuli such as stroke, epilepsy, traumatic spinal cord injury, and traumatic brain injury [19–22]. In addition, our data showed that LPS preconditioning results in a reduced, but not a blocked, immune response. Importantly, LPS induced-immune tolerance in microglia was associated with improved cognitive impairments and anhedonia. Such a long-term depressed effect of LPS has not been described previously with reports in adult animals. Our results are in line with recent studies indicating that LPS preconditioning blunt microglia proinflammatory response by epigenetic suppression of IL-1*β*, TNF-*α*, and IL-6 gene, as well as shifting the proinflammatory response to the anti-inflammatory reaction and increase in the phagocytic activity [23]. The mechanism might be related to NF-*κ*B p65 inhibition, which is critically involved in neuroinflammation and cognitive impairments [24]. Therefore, microglia memory induced by repeated LPS injections can ameliorate subsequent neuroinflammation, prevent excess damage to the brain in case of recurrent inflammatory stimulation, and consequent cognitive impairments. From a clinical view, suppression of the proinflammatory response may have important benefits especially if it can be achieved while maintaining or even enhancing the innate immune clearance of pathogenic organisms. While many previous studies have demonstrated that endotoxin tolerance protects mice from CLP-induced mortality [25, 26], our study did not show such an effect. One reason for this discrepancy is that the time interval between LPS preconditioning and CLP in our study is significantly longer than previous studies, which can cause mortality changes that make the interpretation of data difficult.

Long-term modification of microglial function has been well demonstrated following immune stimulation across studies. Exposure of pregnant or neonatal animals to inflammatory stimuli such as LPS was found to cause alterations in microglial responses and function in response to a second inflammatory stimulus during adulthood [27, 28]. In particular, increasing evidence has demonstrated that IL-1*β* plays an important role in the modulation of trained immunity [29]. In a recent study, 2–4-month-old mice received a single *i.p.* injection of LPS (0.25 mg/kg) was sufficient to reduce IL-1*β* release in the brain in response to a second systemic LPS injection for up to 32 weeks [23]. In addition, IL-1*β* secreted by activated microglia mediated a transient synaptic deficit associated with memory impairments induced by sepsis [30]. In our study, we showed that the hippocampal expressions of IL-1*β* and IL-6 but not other inflammatory factors were upregulated by sepsis, suggesting IL-1*β* and IL-6 are specifically affected by CLP.

On the other hand, microglial priming or sensitization is associated with an exaggerated inflammatory cytokine response associated with cognitive impairment and depressive-like behaviors [31, 32]. By contrast, treatment with the anti-inflammatory bacterium attenuates stress-induced exaggeration of inflammation and anxiety-like behavior [33]. Although it is not well understood, the observation that microglia display a sustained immune memory might be a consequence of their low replacement rate. In addition, previous studies have demonstrated that IL-10 may contribute to but is not absolutely necessary for the development of LPS tolerance [34]. However, our study shows that microglial memory triggered by LPS stimuli at a lower dose did not increase IL-10 level, suggesting other possible mechanism might be involved. Evidently, further investigations are warranted to elucidate these differences.

In summary, our study provides a better understanding of microglial immune memory and offers a potential therapeutic method for sepsis-associated cognitive impairments, although further work is needed to study individual conditions.

## Data Availability

The datasets used and/or analyzed during the present study are available from the corresponding author on reasonable request.

## Additional Points

*Highlights*. Disrupted immune response is a key feature of many neurodegenerative diseases. LPS injections for three consecutive days induced immune memory. Modulation of immune responses by inflammatory stimuli has protective effects.
